# Semitransparent bandages based on chitosan and extracellular matrix for photochemical tissue bonding

**DOI:** 10.1186/s12938-018-0444-1

**Published:** 2018-01-22

**Authors:** Samuel J. Frost, Damia Mawad, Richard Wuhrer, Simon Myers, Antonio Lauto

**Affiliations:** 1 School of Science and Health, Western Sydney University, Locked Bag 1797, Penrith, NSW 2751 Australia; 2 School of Materials Science and Engineering, UNSW Sydney, Sydney, NSW 2052 Australia; 3 Australian Centre for NanoMedicine and ARC Centre of Excellence in Convergent BioNano Science and Technology, UNSW Sydney, Sydney, NSW 2052 Australia; 4 Centre for Advanced Macromolecular Design, UNSW Sydney, Sydney, NSW 2052 Australia; 5 Advanced Materials Characterization Facility (AMCF), Western Sydney University, Locked Bag 1797, Penrith, NSW 2751 Australia; 6 School of Medicine, Western Sydney University, Locked Bag 1797, Penrith, NSW 2751 Australia; 7 Biomedical Engineering & Neuroscience Research Group, The MARCS Institute, Western Sydney University, Locked Bag 1797, Penrith, NSW 2751 Australia

**Keywords:** Biomaterials, Tissue repair, Lasers, Adhesives, Biopolymers

## Abstract

**Background:**

Extracellular matrices (ECMs) are often used in reconstructive surgery to enhance tissue regeneration and remodeling. Sutures and staples are currently used to fix ECMs to tissue although they can be invasive devices. Other sutureless and less invasive techniques, such as photochemical tissue bonding, cannot be coupled to ECMs because of their intrinsic opacity to light.

**Results:**

We succeeded in fabricating a biocompatible and adhesive device that is based on ovine forestomach matrix (OFM) and a chitosan adhesive. The natural opacity of the OFM has been overcome by adding the adhesive into the matrix that allows for the light to effectively penetrate through it. The OFM-chitosan device is semitransparent (attenuation length ~ 106 µm) and can be photoactivated by green light to bond to tissue. This device does not require sutures or staples and guarantees a bonding strength of ~ 23 kPa.

**Conclusions:**

A new semitransparent and biocompatible bandage has been successfully fabricated and characterized for sutureless tissue bonding.

## Background

Extracellular matrices have been used successfully in a vast array of clinical procedures including hernia repair [1–4], anal fistulas [5], urethral and cardiovascular reconstruction [3, 6]. During these procedures, it has remained common practice to use either sutures or staples to affix the ECM scaffold to tissue. Sutures and staples however, may cause damage to the tissue or the ECM and their application can be challenging during laparoscopic procedures. To overcome these difficulties, other methodologies have been recently explored as alternatives to the current techniques; a very promising one employs either a thin tissue membrane or an ECM that is soaked in a rose bengal solution before applying it to tissue [7]. A green laser then irradiates the ECM, photo-activating the rose bengal at the tissue interface and bonding the matrix to tissue without sutures. The mechanism of photochemical bonding is still under investigation although it is speculated that rose bengal has the ability to produce singlet oxygen upon light irradiation, which promotes crosslinking between the amino groups of collagen in the tissue and ECM [8, 9]. The key advantage of photochemical bonding is that no thermal damage is inflicted to tissue despite the laser irradiation, with tissue temperature remaining below 39 °C [10]. The combination of rose bengal solution and light has been applied successfully in several experimental procedures, including blood vessel [11] and nerve anastomoses [12], cornea [13], tendon [14] and skin repair [15, 16]. In the latter case, a human trial established that superficial closure of skin executed with photochemical bonding resulted in better healing than sutured wounds [16]. A further development of this sutureless technique consists of an adhesive film based on chitosan that contains rose bengal; the adhesive has been tested successfully in conjunction with a green laser to repair peripheral nerves [17–19] or seal small intestinal perforations [20]. Chitosan contains amino groups that crosslink to the tissue amino groups upon light irradiation achieving a bonding strength of ~ 15 kPa [10]. The chitosan-rose bengal adhesive (“rose adhesive”) is of particular interest because its surface can be modified with nanopillars mimicking the anatomical structures of the Gecko foot. These nanostructures have been proved to increase the bonding strength of the chitosan adhesive (~ 21 kPa) when compared to a “flat” adhesive without nanopillars [20, 21]. Despite the advantages of photochemical bonding, a significant drawback arises when a thick ECM is bonded to tissue after soaking it in a rose bengal solution. This is because insufficient light reaches the tissue interface due to the ECM opacity, which prevents photochemical bonding. The ECMs used in most of the surgical procedures highlighted above are multilayered and thus opaque; unless this opacity is eliminated, photochemical tissue bonding remains a non-viable alternative to sutures and staples. In the present investigation, we solved the opacity problem by fabricating a sutureless and semitransparent ECM that incorporates the rose adhesive; this new device is capable of photo-bonding to tissue with a remarkable strength of ~ 23 kPa.

## Methods

### Bandage and adhesive fabrication

**Fig. 1:**
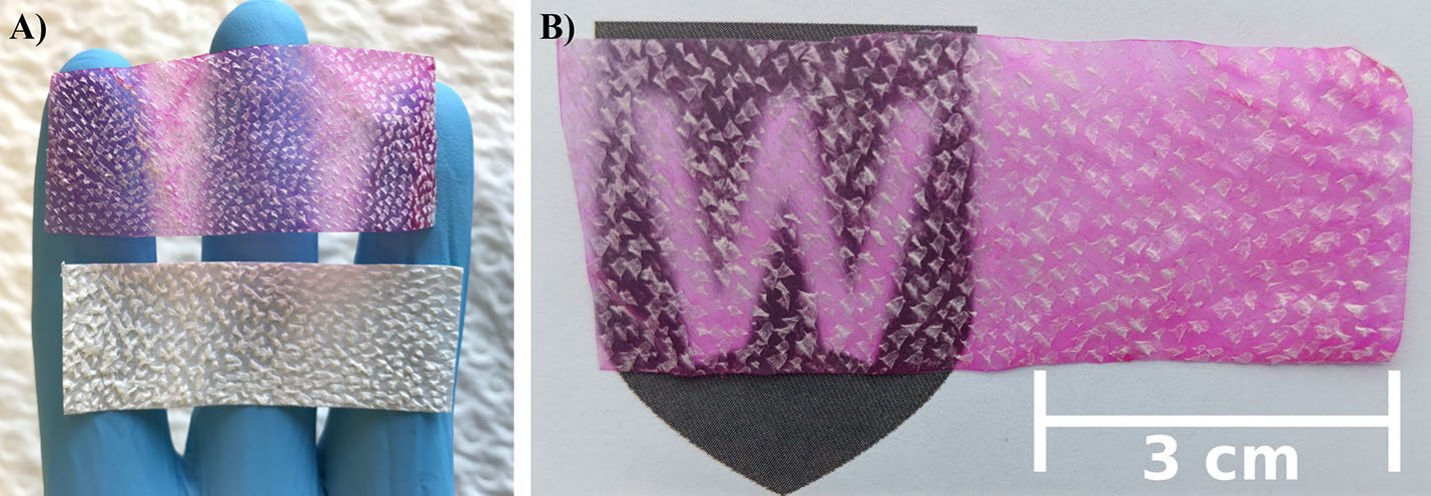
The dry bandage (top) is compared to the dry OFM (bottom) in (**A**); integration of the chitosan adhesive inside the extracellular matrix transformed the opaque OFM into a semitransparent matrix. The transparency of the bandage is remarked in (**B**)

All chemicals were purchased from Sigma-Aldrich (Sydney, Australia) and were used without any further purification. An in-depth description of the adhesive preparation has been already published [22]. Briefly, deacetylated chitosan (≥ 80%, MMW) was dissolved at a concentration of 1.7% w/v in deionised water (50 mL) containing acetic acid (2% w/v) and rose bengal (RB, 0.01% w/v). The solution was stirred for 14 days at room temperature shielded from light to avoid photo-bleaching of the RB. The resultant solution was then centrifuged at 3270 g for 1 h to eliminate impurities. Using a sterile syringe, the supernatant was subsequently spread evenly on a sterile Perspex plate at a ratio of 1 mL to 12 cm^2^ and allowed to dry for ~ 14 days at room temperature in the dark. The resultant “rose adhesive” film was insoluble in water; the adhesive was cut into strips (~ 6 × 10 mm^2^) and the thickness was recorded using a digital micrometer (Model 293-831, Mitutoyo, Japan). The strips were then placed between sterile glass slides to preserve shape and stored in the dark at room temperature. For the bandage fabrication, the ovine forestomach matrix (OFM, Aroa Biosurgery, Auckland, New Zealand) was cut into sections measuring ~ 3 × 7 cm^2^. A sterile syringe was then used to dispense ~ 1.8 mL of rose adhesive on a Perspex plate over an area of 21 cm^2^ to match the dimensions of the OFM. The OFM sections were then carefully placed over the rose adhesive using a pair of forceps to ensure no air bubbles and even contact at the interface. The sections were then left for ~ 14 days in the dark at room temperature to allow the adhesive solution to impregnate the OFM and dry. The newly fabricated OFM-rose adhesive, hereafter referred to as the “bandage”, appeared semitransparent if compared to the opaque OFM (Fig. 1). The bandage was then cut into smaller rectangular strips and their dimensions recorded; the digital micrometer was used to measure the thickness.

### Optical attenuation

The optical attenuation of the bandage, rose adhesive and OFM were measured at 532 nm using a Shimadzu UV-1800 spectrophotometer. This wavelength is strongly absorbed by rose bengal and corresponds to the laser wavelength used for the tissue repair [23]. The samples were soaked in deionised water for 30 s and individually mounted in plastic cuvettes ensuring uniform flatness. Spectra were then recorded for each group in the range of 400–800 nm. Assuming the validity of Beer’s law, the attenuation was calculated using:

$$ I = I_{0} e^{ - Ax} $$

where *I*_0_ is the incident beam intensity, 1/*A* is the attenuation length, and *x* is the thickness of the sample.

### In vitro model for photochemical tissue bonding

**Fig. 2:**
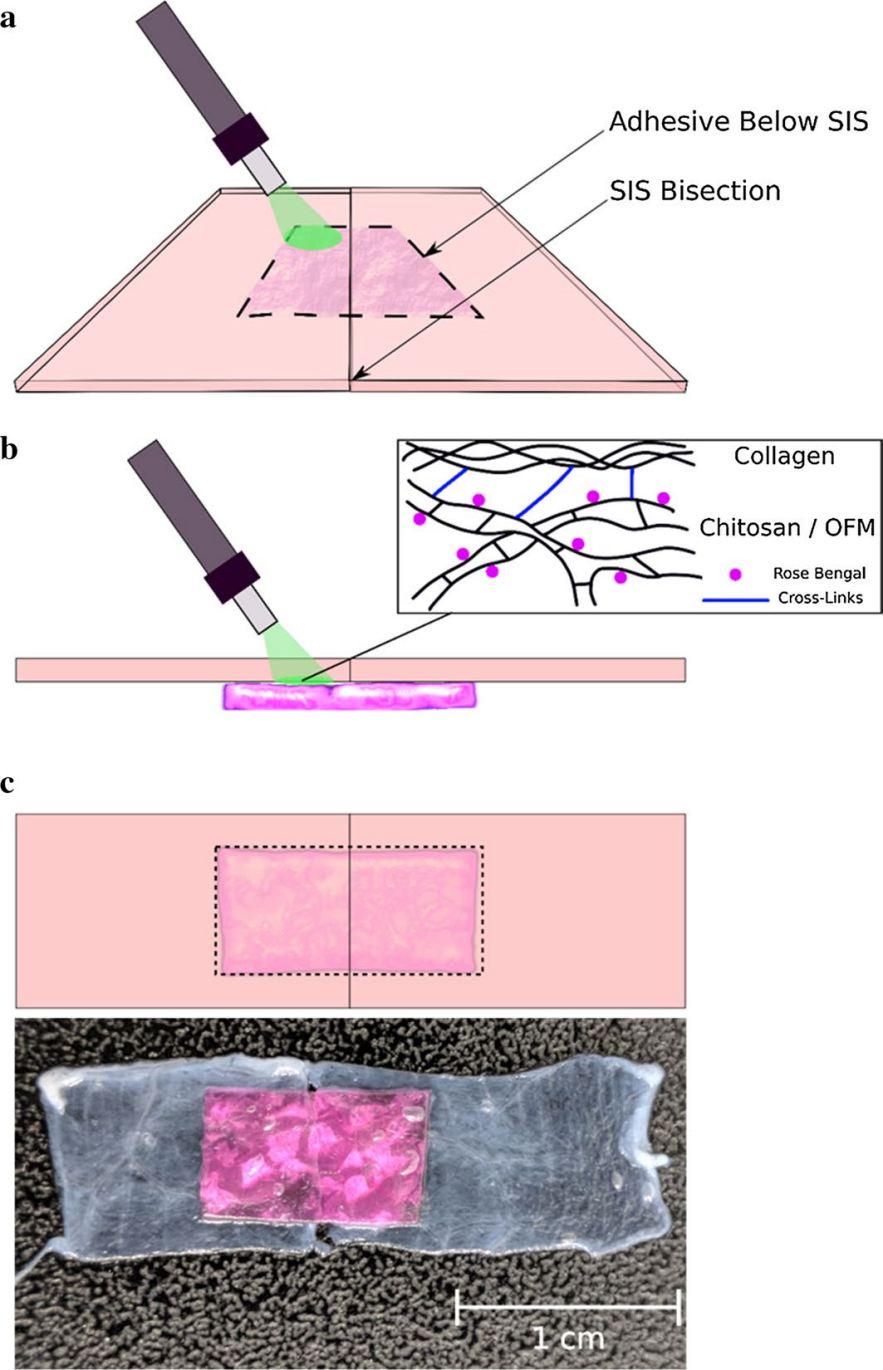
Schematic of photochemical tissue bonding on small intestine submucosa (SIS). **a** The adhesive device is placed underneath the bisected SIS and is laser-irradiated through the transparent SIS. **b** At the tissue interface, the laser is absorbed by rose bengal generating singlet oxygen, which in turn facilitates the crosslinking of tissue collagen and chitosan via amino groups. **c** The photochemical tissue bonding results in a strong repair that reconnects the bisected sections of SIS

The adhesive strength of the photo-activated bandage, rose adhesive and OFM were tested in vitro on sections of ovine small intestinal submucosa (SIS) that was prepared following the report by Badylak et al. [24]. Briefly, following euthanasia, sections of small intestine (~ 15 cm) were immediately harvested from the animal, cleaned and stored at − 80 °C. Prior to use, the sections were immersed in deionised water to thaw for approximately 15 min. The small intestine sections were then washed thoroughly with water and all mesenteric tissues were excised [24, 25]. The sections were then carefully turned inside out before mechanically removing the epithelium and muscularis mucosae. Finally, after being turned back into its original shape the muscularis externa and serosa were removed resulting in a semi-transparent tube. The SIS tube was then cut into rectangular sections (~ 1 × 2 cm^2^) prior to the laser repair procedure (Fig. 2).

### Laser tissue repair

Tissue was repaired using the bandage, rose adhesive and OFM to assess and compare their bonding strength. A solid state diode-pumped laser was used during the procedures, the laser emitted a power of 180 mW in continuous wave at 532 nm through a multimode fiber (core diameter = 200 µm) and with a spot size of ~ 5 mm (Model MGL-W532, CNI Lasers, China).

#### Group 1 (bandage)

**Fig. 3:**
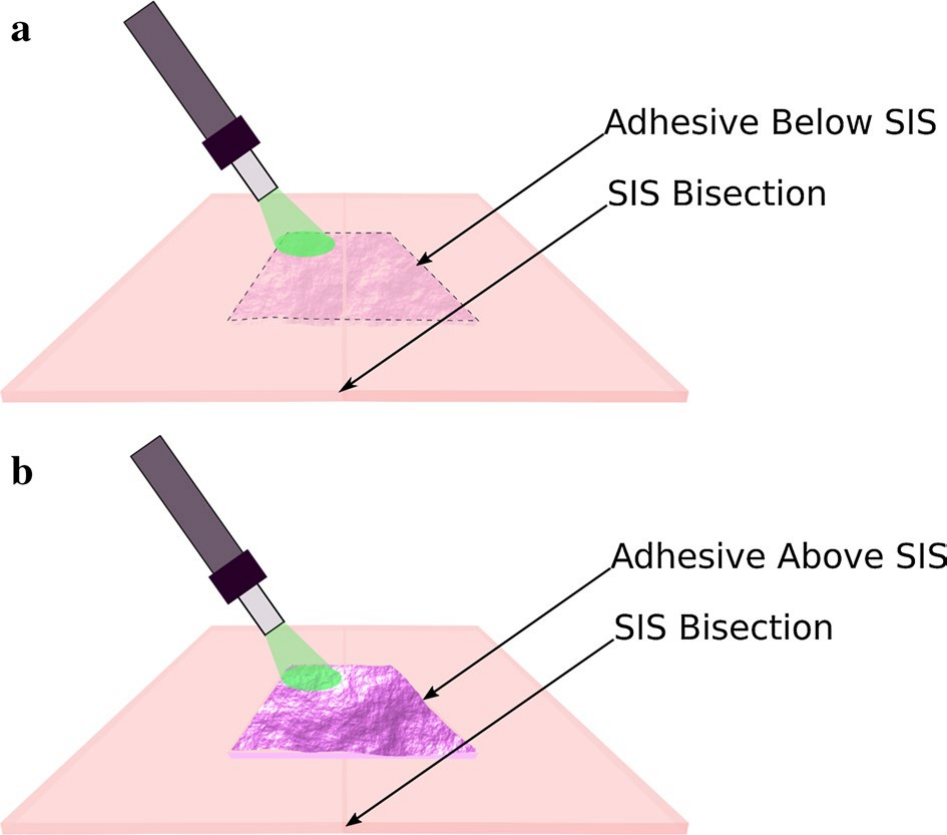
The adhesive device is placed under the SIS (thickness = 45 ± 5 µm) to standardize the amount of light reaching the tissue interface (**a**). When the bandage is positioned on the top of the SIS (**b**), light is attenuated more at the tissue interface because of the large thickness of the bandage (133 ± 17 µm)

The SIS was bisected and approximated end-to-end under an operating microscope; the bandage was then placed across the bisection line with forceps, adhering to tissue. The SIS with the bandage was carefully turned upside-down with forceps making sure the whole sample remained intact (Fig. 3a). The bandage was then spot-irradiated through the SIS with the green laser ensuring ~ 5 s irradiation on each spot; this resulted in multiple passes over the sample and a total irradiation time of ~ 6 min. The irradiation of the bandage placed under the SIS is very convenient as the laser beam is attenuated only by the SIS before reaching the bandage interface, where bonding occurs. This ensures the fluence delivered to each sample is fairly constant at the tissue interface, being subjected only to the minimal fluctuation (~ 3 µm) of the SIS thickness. When the bandage is positioned above the SIS, the laser attenuation at the tissue interface depends instead on the bandage thickness that has a large variation from sample to sample (~ 14 µm). A summary of the laser parameters can be found in Table 1.

**Table 1:** Laser parameters

Power (mW)	Time (s)	Fluence (J/cm^2^)	Irradiance (W/cm^2^)	Spot Size (mm)
180 ± 5	366 ± 5	~ 110	~ 0.9	~ 5

*Power* laser power (mean ± maximum error), *Time* irradiation time (mean ± maximum error), *Fluence* average laser fluence, *Irradiance* estimated irradiance

#### Group 2 (rose adhesive)

The procedure adopted was identical to the one described in *Group 1*, although the rose adhesive was used instead of the bandage.

#### Group 3 (OFM)

The SIS was bisected and approximated end-to-end under the operating microscope, the OFM was then soaked in a deionised water solution of rose bengal (0.1% w/v) for ~ 60 s as described by Fairbairn et al. [7], and placed over the SIS bisection line. The SIS with the OFM was carefully turned upside-down with forceps and the OFM spot-irradiated through the SIS, as described in *Group 1*.

#### Group 4 (bandage on the top)

The procedure adopted was identical to the one described in *Group 1*; in this instance though the bandage was left on the top of the SIS without turning the whole sample upside-down to allow direct irradiation of the bandage (Fig. 3b).

### Mechanical testing

To determine the strength of the repair, each sample was tested using a single column tensiometer (Instron 3343, Instron, Massachusetts, USA) interfaced with a personal computer. The tissue was kept hydrated after irradiation prior to testing to mimic in vivo conditions. The ends of the SIS with the repair were placed into the grips and separated at 22 mm/min until the sample separated at a maximum load drop of 80%. The recorded strength was used to calculate the repair strength by dividing the maximum load (N) by the adhesive surface area [26]. Additional mechanical tests were performed on the rose adhesive, OFM strips, and fabricated bandages to assess tensile strength, Young’s modulus and elongation. Samples (~ 4 × 16 mm^2^) were tested either dry or wet using 100 µL of deionised water 1 min prior to the commencement of the test, in order to mimic physiological conditions. Samples were separated at a rate of 22 mm/min until a cohesive failure was recorded.

### Scanning electron microscopy (SEM)

Scanning electron microscopy was used to image the microstructure of the matrix after the bandage fabrication. Samples (n = 5) were then cut into sections (~ 1 × 2 mm^2^) and washed with deionised water before being fixed in Karnovsky’s solution (2.5% paraformaldehyde and 2% gluteraldehyde) and dehydrated in ethanol at increasing dilutions of 30, 50 and 75%. The samples were imaged with a scanning electron microscope (model JEOL JSM 6510LV, JEOL, Japan) in low vacuum mode at 30 Pa and 20 kV accelerating voltage, using the back-scattered electron detector. A working distance of 15 mm was used for imaging.

### Statistical analysis

Data analysis was performed using the unpaired two-tailed t test, ANOVA one-way and Tukey’s post-test at a significance level of 0.05. Values are reported as mean ± standard deviation.

## Results

### Bandage fabrication and optical attenuation

**Fig. 4:**
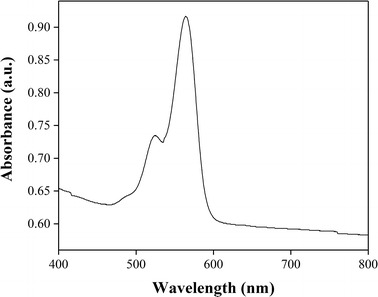
Absorption spectrum of the bandage in the 400–800 nm range. The bandage retains the typical absorption peaks of rose bengal around 524 and 564 nm

**Fig. 5:**
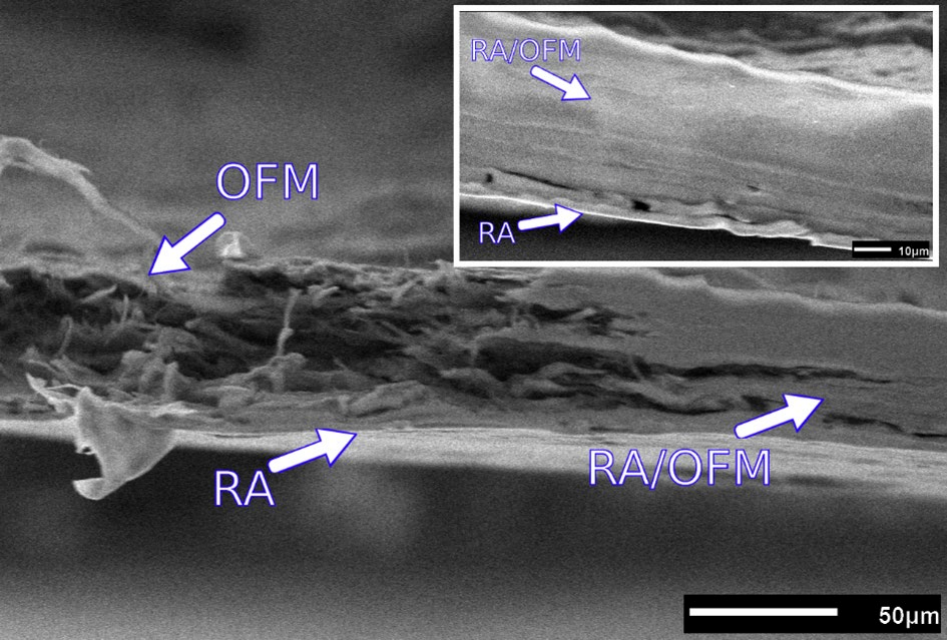
SEM image of the bandage, on the left side the OFM structure is visible while on the right the rose adhesive is integrated throughout the matrix thickness (RA/OFM). A very thin film of rose adhesive (~ 5 µm) is also layered on the bottom of the bandage (inset image)

During the fabrication stage, the OFM material became semi-transparent upon integration with the rose adhesive and light could be seen through the bandage even with the naked eye (Fig. 1). This was a remarkable outcome considering that the opacity of the OFM alone (without rose adhesive) or impregnated with the rose bengal solution made impossible to measure its attenuation length. The water in the integrated adhesive is mostly responsible for the semi-transparency of the bandages: light is guided inside the bandage because of the adhesive that is a denser medium than air. Wet bandages also had a higher attenuation length compared to the dry ones (106 ± 11 and 68 ± 8 µm respectively). The wet rose adhesive was likewise more transparent than the dry adhesives; a summary of the attenuation lengths is given in Fig. 4 and Table 2. SEM imaging confirmed that the adhesive was incorporated into the native OFM structure throughout its thickness (Fig. 5). Notably, a thin layer of rose adhesive (5 ± 2 μm, n = 5) was also formed on the underside of the bandage. This adhesive layer is essential for allowing tissue bonding in conjunction with a laser.

**Table 2:** Attenuation length summary

n = 10	Dry rose adhesive	Wet rose adhesive	Dry bandage	Wet bandage	SIS
Attenuation length (μm)	23 ± 1	86 ± 9*	68 ± 8	106 ± 11^♦^	33 ± 3
Thickness (µm)	11 ± 1	24 ± 2	116 ± 15	133 ± 17	44 ± 5

All values are given as mean ± standard deviation (n = 10); the suffixes indicates statistical difference between dry and wet samples (two-tails paired t test, *p = 1.48E−9, ^♦^p = 8.40E−6). The optical density of OFMs alone or impregnated with rose bengal solutions could not be measured because of their opacity

### Laser tissue repair

**Fig. 6:**
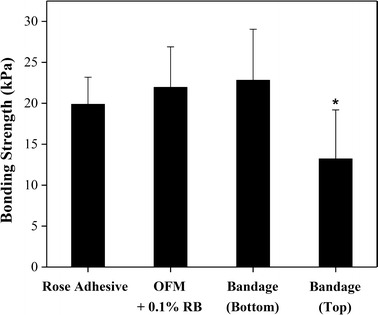
The bonding strengths of the bandage, rose adhesive and rose bengal + laser technique are not significantly different. When the bandage is placed on the top of tissue, the bonding strength decreases because less light reaches the tissue interface to activate photochemical bonding (ANOVA 1-way, p < 0.0001, n = 30)

The bandage bonded successfully to the SIS after laser irradiation, achieving a bonding strength of 22.8 ± 6.2 kPa (Fig. 6). This result shows that the semitransparent bandage can effectively be attached to tissue if coupled with a green laser. The other methods of tissue bonding tested in our study were as strong as the bandage; soaking the OFM in a rose bengal solution, for example, resulted in a bonding strength of 22.0 ± 4.9 kPa while the rose adhesive strength was 19.9 ± 3.3 kPa (ANOVA one-way, p = 0.0684, n = 30). When the bandage was placed on top of the SIS and irradiated by the laser (“*Bandage on the Top”* group) the bonding strength was lower than the other three groups (13.2 ± 6.0 kPa, ANOVA one-way, p < 0.0001, n = 30). In this case, the laser is attenuated more at the tissue interface because the light travels through the bandage which has a larger thickness than the SIS. Nevertheless, this bonding strength is still effective and comparable to the strength of the rose adhesive (~ 15 kPa) that was used to successfully repair peripheral nerves in vivo [18]; in that instance the adhesive was also positioned on the top of the nerve and irradiated thereafter. When the matrix was impregnated with rose bengal solution and irradiated on the top of tissue, no bonding occurred even if the fluence and power were increased up to 220 J/cm^2^ and 360 mW; it was noted that the matrix had partially melted at this power level. This outcome indicates that a thick extracellular matrix is not suitable for tissue bonding because of its opacity.

The key advantage of our SIS model is the capability of testing with consistency the bonding strength of different adhesive devices (bandage, OFM, adhesive): positioning the device under the SIS ensures that the laser is attenuated similarly at the tissue interface in all groups as the SIS thickness is the only attenuation barrier. All the bandage and OFM samples detached from tissue without breaking in 2 parts (surface failure); the majority (73%) of adhesive samples had also a surface failure while the other samples (27%) failed cohesively.

### Mechanical testing

**Fig. 7:**
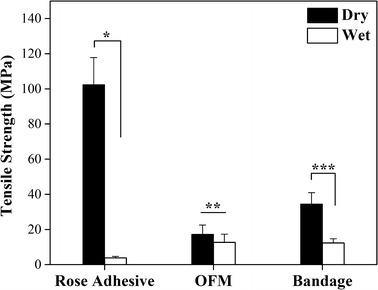
Column representation of the tensile strength for bandage, OFM and rose adhesive in dry and wet conditions (two-tails unpaired t test, *p = 2.54E−25, **p = 0.0008, ***p = 2.04E−19). The tensile strength of the wet bandage is not significantly different from the OFM. The tensile strength of the wet adhesive is significantly lower than the bandage and OFM (ANOVA 1-way, p = 0.0017)

**Fig. 8:**
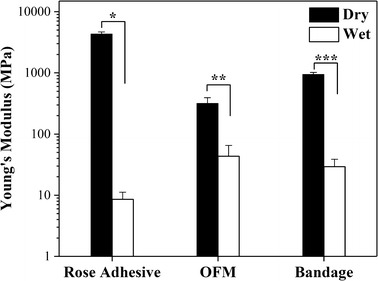
Column representation of the Young’s modulus (logarithmic scale); there was a decrease of stiffness in the wet devices if compared to the dry ones (two-tails unpaired t-test, *p = 7.57E−33, **p = 2.67E−19, ***p = 2.55E−34). The Young’s modulus of the wet bandages was not statistically different from the wet OFM, implying that the bandage stiffness is mostly due to the OFM structure

**Fig. 9:**
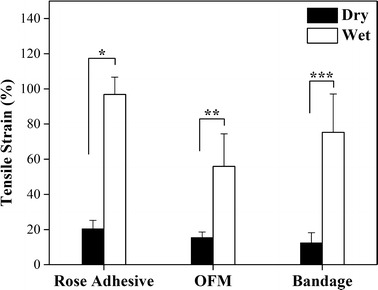
The tensile strain of the biodevices increased significantly when they were wet (two-tails unpaired t test, *p = 2.98E−34, **p = 5.90E−13, ***p = 1.48E−16). The tensile strain of wet bandages was between the adhesive and OFM tensile strain, indicating that both the OFM and integrated adhesive contributed to the bandage strain

A considerable change in mechanical properties was observed in the bandage after hydration (Table 3). There was a significant decrease between the tensile strength and Young’s modulus of dry and wet bandages (Figs. 7 and 8, respectively). The tensile strain of the wet bandage on the other hand increased, as seen in Fig. 9 (two-tails unpaired t test, p < 0.05, n = 30). It was noted that the thickness of wet bandages increased ~ 14% more than the dry ones (n = 20) because of water uptake, which is an important factor in changing mechanical properties. The elongation capability is enhanced by the hydrogen bonds between the polymer chains and water inside the bandages, while the stiffness is diminished. The mechanical properties of the OFM and adhesive changed similarly when they were wet, as illustrated in Table 3. It was observed that the thickness of the wet rose adhesive increased ~ 110% more than the dry adhesive (n = 20) while the thickness of the OFM measured ~ 26% less than the dry OFM (n = 40). In the latter case, there is an apparent contradiction: the OFM clearly uptakes water as indicated by the sharp decrease in stiffness and strain increase, on the other hand the thickness seemed reduced. This reduction is likely due to the collapse of the rigid multilayer structure of the OFM when wet. The bandage tensile strength was not significantly different to that of the OFM (~ 12 MPa) when wet; in this case the contribution of the adhesive to the bandage tensile strength is secondary to the OFM contribution. The stiffness of the bandage is also due to the OFM stiffness while the tensile strain (75%) of the bandage is midway between the adhesive and OFM tensile stains (97 and 56%, respectively).

**Table 3:** Summary of mechanical properties (n = 30)

n = 30	Max load (N)	Tensile strength (MPa)	Young’s modulus (MPa)	Tensile strain (%)	Length (mm)	Width (mm)	Thickness (µm)
Dry rose adhesive	6.6 ± 1.7	(1.02 ± 0.15)*10^2^	(4.30 ± 0.37)*10^3^	20 ± 5	15 ± 1	4.4 ± 0.5	15 ± 2
Wet rose adhesive	0.6 ± 0.2	3.8 ± 0.8	8.6 ± 2.6	97 ± 10	15 ± 2	5.1 ± 0.9	32 ± 7
Dry OFM	14.1 ± 5.2	17.2 ± 5.3	316 ± 77	15 ± 3	15 ± 1	4.6 ± 0.7	177 ± 27
Wet OFM	7.9 ± 2.5	12.7 ± 4.7	43 ± 22	56 ± 19	16 ± 1	4.8 ± 0.7	134 ± 22
Dry bandage	16.1 ± 5.2	34.4 ± 6.5	944 ± 73	12 ± 6	16 ± 1	3.7 ± 0.3	127 ± 24
Wet bandage	5.9 ± 1.3	12.3 ± 2.4	29 ± 9	75 ± 22	16 ± 1	3.4 ± 0.4	142 ± 23

## Discussion

The clinical use of extracellular matrices is widespread, nonetheless the current methods of attachment (sutures and staples) can be problematic in several surgical procedures. Our study details the fabrication and in vitro application of a semitransparent extracellular matrix, which is bonded to tissue by a low power laser, exploiting photochemical bonding and thus avoiding any thermal damage [10, 27]. Light is a convenient trigger for the bandage because it is sterile and activates adhesion whenever the surgeon requires it. More remarkably, this technology relies on a chitosan adhesive film and rose bengal for tissue bonding and does not use sutures or staples that are notoriously invasive for the host tissue. Laser-activated chitosan adhesive films have been successfully developed and tested in rats by our group to repair peripheral nerves [17, 18], which had better functional recovery than nerves repaired with sutures 3 months post-operatively [19]. These nerves were also free from any macroscopic signs of neuroma or significant inflammation. Another significant application of the adhesive technology demonstrated that an electrically conductive patch could be laser-bonded to rat hearts using the rose adhesive. The patch was partially coated with a conducting polymer (polyaniline) to allow current flow. In vivo experiments showed that the patch was firmly attached to tissue and did not induce proarrhythmogenic activities in the heart or caused significant inflammation 2 weeks after implantation [28]. Other sutureless techniques for tissue repair include bioglues that may have detrimental side effects; cyanoacrylate-based glues for example can induce toxicity and trigger adhesion prematurely because of their high reactivity [29]. Glues that are in liquid or gel form often present issues, as the surface of the tissue must be dry and clear of physiological fluids to avoid unwanted dilution [30].

Our study shows that coupling light with an extracellular matrix is possible because of the integrated chitosan adhesive that transformed the opaque matrix into a semitransparent bandage. When light enters from one side of the thin rectangular bandage, it is guided inside as the integrated adhesive is a denser medium than air. The guided light escapes mostly from the opposite side (~ 21 cm^2^) of the bandage rather than from the lateral sides, which are very small (bandage thickness ~ 140 μm). Other recent studies have reported similar light-guiding properties in polymeric films [31]. The extracellular matrix without adhesive is unable to guide light, which is instead scattered at the surface causing the ECM opacity. When the bandage is wet there is an extra uptake of water that has the effect of diluting the rose bengal concentration and further enhance the transparency at 532 nm.

Another method for applying extracellular matrices to tissue without sutures is by soaking them in a rose bengal solution before irradiation [7]. This modality has the advantage of being simple as it does not require the fabrication of the adhesive and bandage; on the other hand, the transparency of the matrix can be a significant issue that relegates this method only to thin matrices. It was indeed impossible to perform photochemical bonding in this study when the soaked OFM was applied on the top of tissue. Our group attempted to bond the OFM on the top of tissue using fluences up to ~ 220 J/cm^2^ (power = 360 mW) but no bond occurred and the matrix melted.

The comparison of the bandage performance with the “soaking” method and the bare adhesive is important to establish advantages and disadvantages associated with these techniques. The major obstacle for assessing and comparing their bonding strengths is to standardize the amount of light reaching the tissue interface, where the photochemical tissue bonding occurs. Placing the adhesive device on the top of tissue has the significant disadvantage of gathering light at the tissue interface after light has crossed the device thickness. This is unfortunate as the bandage has a highly variable thickness that is also significantly different from the thickness of the extracellular matrix and adhesive. This problem was solved by placing the devices under a thin tissue section (SIS), which was prepared for this particular application. The SIS is translucent, very thin (~ 45 μm) and has a small thickness variation of ~ 10% (Table 2), ensuring that a consistent amount of light reaches the tissues interface of every adhesive device. The outcome of our study shows that the three methodologies of tissue bonding are not statistically different, the bonding strength being ~ 20 kPa. The bandage and adhesive bond to tissue with the same strength of the extracellular matrix soaked in rose bengal; it is noted that both the bare adhesive and bandage have a layer of chitosan film in contact with tissue (SIS) that explains the equivalent bonding strength. When the bandage is placed on the top of the tissue, the bonding strength is still effective although decreased (~ 13 kPa) as less light reaches the tissue interface where photochemical reactions take place and bonding is formed [8, 9]. The bonding strength of the bandage on the top is comparable to the strength reported in previous studies where peripheral nerves and intestine were successfully repaired either in vitro or in vivo [18–20]. Bandages have very similar mechanical properties to their OFM component and are therefore suitable for surgical implantation. The thickness uncertainty of the OFM, due to natural variability, determined a relatively large standard deviation of the bonding strength, tensile strength and Young’s modulus of bandages; statistical tests could however determine differences among the experimental groups. The OFM used in our experiments is naturally larger and stronger [32] than many extracellular matrices currently used in clinical practice [33]. This OFM is particularly suited for surgical applications because it promotes cell proliferation and angiogenesis in vitro; previous studies have also shown that it increases blood vessel density when compared to ECMs derived from small intestinal submucosa [34, 35]. Of note is the degradability of the rose adhesive inside the bandage; a recent study showed that when a physiological amount of lysozyme is added to the adhesive, its depolymerization is significantly accelerated over a period of 1–4 weeks [36]. It is indeed important that cells migrate inside the extracellular matrix of the bandage to remodel tissue and enhance regeneration [37]. Another strategy for allowing cell migration inside the OFM is the integration of the adhesive only in a limited portion of the bandage. Covering 15% of the bandage area with the adhesive, for example, guarantees a strong tissue bonding that can withstand a pulling force of ~ 5 N while most of the bandage surface is available for cell interaction.

## Conclusions

In conclusion, we have fabricated and characterized a semitransparent and biocompatible bandage for sutureless tissue fixation. The bandage comprises an extracellular matrix with an integrated chitosan adhesive that is activated photochemically by green light; both components being non-toxic and biocompatible. Key for the semi-transparency of the bandage is the incorporation of rose adhesive into the opaque matrix that allows for the light to effectively penetrate through it. Secondary to this we have also devised an effective method of comparing the bonding strengths of different adhesive devices, regardless of composition, directly at the interface of the tissue by irradiating through a transparent layer of freshly prepared ovine SIS.

## Abbreviations

ECMs: extracellular matrices
MMW: medium molecular weight
OFM: ovine forestomach matrix
RB: rose bengal
SIS: small intestinal submucosa

## References

[1] Catena F, Ansaloni L, Leone A, De Cataldis A, Gagliardi S, Gazzotti F (2005). Lichtenstein repair of inguinal hernia with Surgisis inguinal hernia matrix soft-tissue graft in immunodepressed patients. Hernia.

[2] Chung W, Kazemi P, Ko D, Sun C, Brown CJ, Raval M (2009). Anal fistula plug and fibrin glue versus conventional treatment in repair of complex anal fistulas. Am J Surg.

[3] Witt RG, Raff G, Van Gundy J, Rodgers-Ohlau M, Si M-S (2013). Short-term experience of porcine small intestinal submucosa patches in paediatric cardiovascular surgery. Eur J Cardiothorac Surg.

[4] Katkhouda N (2004). A new technique for laparoscopic hernia repair using fibrin sealant. Surg Technol Int.

[5] Ky AJ, Sylla P, Steinhagen R, Steinhagen E, Khaitov S, Ly EK (2008). Collagen fistula plug for the treatment of anal fistulas. Dis Colon Rectum.

[6] Fiala R, Vidlar A, Vrtal R, Belej K, Student V (2007). Porcine small intestinal submucosa graft for repair of anterior urethral strictures. Eur Urol.

[7] Fairbairn NG, Ng-Glazier J, Meppelink AM, Randolph MA, Valerio IL, Fleming ME (2015). Light-activated sealing of nerve graft coaptation sites improves outcome following large gap peripheral nerve injury. Plast Reconstr Surg.

[8] Au V, Madison SA (2000). Effects of singlet oxygen on the extracellular matrix protein collagen: oxidation of the collagen crosslink histidinohydroxylysinonorleucine and histidine. Arch Biochem Biophys.

[9] Encinas MV, Rufs AM, Bertolotti SG, Previtali CM (2009). Xanthene dyes/amine as photoinitiators of radical polymerization: a comparative and photochemical study in aqueous medium. Polymer.

[10] Lauto A, Mawad D, Barton M, Gupta A, Piller SC, Hook J (2010). Photochemical tissue bonding with chitosan adhesive films. Biomed Eng Online.

[11] O’Neill AC, Winograd JM, Zeballos JL, Johnson TS, Randolph MA, Bujold KE (2007). Microvascular anastomosis using a photochemical tissue bonding technique. Lasers Surg Med.

[12] Johnson TS, O’Neill AC, Motarjem PM, Amann C, Nguyen T, Randolph MA (2007). Photochemical tissue bonding: a promising technique for peripheral nerve repair. J Surg Res.

[13] Mulroy L, Kim J, Wu I, Scharper P, Melki SA, Azar DT (2000). Photochemical keratodesmos for repair of lamellar corneal incisions. Invest Ophthalmol Vis Sci.

[14] Chan BP, Amann C, Yaroslavsky AN, Title C, Smink D, Zarins B (2005). Photochemical repair of Achilles tendon rupture in a rat model1. J Surg Res.

[15] Xu N, Yao M, Farinelli W, Hajjarian Z, Wang Y, Redmond RW (2015). Light-activated sealing of skin wounds. Lasers Surg Med.

[16] Tsao S, Yao M, Tsao H, Henry FP, Zhao Y, Kochevar JJ (2012). Light-activated tissue bonding for excisional wound closure: a split-lesion clinical trial. Br J Dermatol.

[17] Lauto A, Foster LJ, Avolio A, Sampson D, Raston C, Sarris M (2008). Sutureless nerve repair with laser-activated chitosan adhesive: a pilot in vivo study. Photomed Laser Surg.

[18] Barton M, Morley JW, Stoodley MA, Ng K-S, Piller SC, Duong H (2013). Laser-activated adhesive films for sutureless median nerve anastomosis. J Biophotonics.

[19] Barton MJ, Morley JW, Stoodley MA, Shaikh S, Mahns DA, Lauto A (2015). Long term recovery of median nerve repair using laser-activated chitosan adhesive films. J Biophotonics.

[20] Frost SJ, Mawad D, Higgins MJ, Ruprai H, Kuchel R, Tilley RD (2016). Gecko-inspired chitosan adhesive for tissue repair. NPG Asia Mater.

[21] Frost SJ, Mawad D, Hook J, Lauto A (2015). Micro- and Nanostructured Biomaterials for Sutureless Tissue Repair. Adv Healthc Mater.

[22] Lauto A, Stoodley M, Barton M, Morley JW, Mahns DA, Longo L (2012). Fabrication and application of rose bengal-chitosan films in laser tissue repair. J Vis Exp JoVE.

[23] Neckers DC (1989). Rose Bengal. J Photochem Photobiol Chem.

[24] Badylak SF, Tullius R, Kokini K, Shelbourne KD, Klootwyk T, Voytik SL (1995). The use of xenogeneic small intestinal submucosa as a biomaterial for Achille’s tendon repair in a dog model. J Biomed Mater Res.

[25] Yi J-S, Lee H-J, Lee H-J, Lee I-W, Yang J-H (2013). Rat peripheral nerve regeneration using nerve guidance channel by porcine small intestinal submucosa. J Korean Neurosurg Soc.

[26] Barton MJ, Morley JW, Mahns DA, Mawad D, Wuhrer R, Fania D (2014). Tissue repair strength using chitosan adhesives with different physical-chemical characteristics. J Biophotonics.

[27] Barton M, Piller SC, Mahns DA, Morley JW, Mawad D, Longo L (2012). In vitro cell compatibility study of rose bengal—chitosan adhesives. Lasers Surg Med.

[28] Mawad D, Mansfield C, Lauto A, Perbellini F, Nelson GW, Tonkin J (2016). A conducting polymer with enhanced electronic stability applied in cardiac models. Sci Adv.

[29] Montanaro L, Arciola C, Cenni E, Ciapetti G, Savioli F, Filippini F (2000). Cytotoxicity, blood compatibility and antimicrobial activity of two cyanoacrylate glues for surgical use. Biomaterials.

[30] Bernie JE, Ng J, Bargman V, Gardner T, Cheng L, Sundaram CP (2005). Evaluation of hydrogel tissue sealant in porcine laparoscopic partial-nephrectomy model. J Endourol.

[31] Nizamoglu S, Gather MC, Humar M, Choi M, Kim S, Kim KS (2016). Bioabsorbable polymer optical waveguides for deep-tissue photomedicine. Nat Commun.

[32] Floden EW, Malak SFF, Basil-Jones MM, Negron L, Fisher JN, Lun S (2011). Biophysical characterization of ovine forestomach extracellular matrix biomaterials. J Biomed Mater Res B Appl Biomater.

[33] Andree B, Bar A, Haverich A, Hilfiker A (2013). Small intestinal submucosa segments as matrix for tissue engineering: review. Tissue Eng. Part B Rev.

[34] Lun S, Irvine SM, Johnson KD, Fisher NJ, Floden EW, Negron L (2010). A functional extracellular matrix biomaterial derived from ovine forestomach. Biomaterials.

[35] Irvine SM, Cayzer J, Todd EM, Lun S, Floden EW, Negron L (2011). Quantification of in vitro and in vivo angiogenesis stimulated by ovine forestomach matrix biomaterial. Biomaterials.

[36] Mawad D, Warren C, Barton M, Mahns D, Morley J, Pham BT (2015). Lysozyme depolymerization of photo-activated chitosan adhesive films. Carbohydr Polym.

[37] Lauto A (2009). Integration of extracellular matrix with chitosan adhesive film for sutureless tissue fixation. Lasers Surg Med.

